# Near-Infrared Responsive Nanoplatform for Synergistic Photothermal Therapy/Chemotherapy of Lung Cancer

**DOI:** 10.34133/bmr.0319

**Published:** 2026-03-06

**Authors:** Kangqi Ren, Jian Wang, Lei Xue, Jieyu Liu, Jun Li, Degang Liu, Qiang Yang, Jiangzhou Peng

**Affiliations:** ^1^ Department of Thoracic Surgery, The Shenzhen People’s Hospital, The Second Clinical Medical College of Jinan University, Shenzhen 518020, China.; ^2^ Department of Thoracic Surgery, The Third Affiliated Hospital of Southern Medical University, Guangzhou 510630, China.

## Abstract

Lung cancer is one of the most prevalent and lethal solid tumors worldwide. Traditional treatment methods mainly rely on chemotherapeutic drugs, which kill tumor cells by inhibiting their mitosis and proliferation. However, in clinical practice, first-line chemotherapeutic agents often exhibit short half-lives and induce a range of adverse reactions while potentially leading to tumor cell resistance, significantly reducing therapeutic efficacy. To address these issues, we developed a novel nanoplatform (TSPD), which used titanium dioxide (TiO_2_) as a core template to fabricate porous silica-coated nanoparticles (TiO_2_@SiO_2_), further coated with the photothermal agent polydopamine (PDA) and loaded with docetaxel (DTX). Once internalized by tumor cells, TSPD rapidly released DTX under the acidic conditions of the endosomes, effectively inhibiting tumor cell proliferation. Upon near-infrared (NIR) irradiation, TSPD induced localized hyperthermia, which not only promoted tumor cell death but also accelerated the release of DTX. Furthermore, TSPD exhibited a prolonged tumor retention in vivo, markedly increasing drug retention time at the tumor site, thus enhancing the therapeutic effect. This study provides a promising strategy for the development of novel nanomedicines in the synergistic application of photothermal therapy (PTT) and chemotherapy.

## Introduction

Lung cancer is one of the most prevalent and deadly solid tumors globally, posing a severe threat to human health and life [1,2]. Traditional treatment methods mainly rely on chemotherapeutic drugs, which kill tumor cells by inhibiting their mitosis and proliferation [3–5]. Docetaxel (DTX), a semi-synthetic analog of paclitaxel (PTX), is a potent antimitotic agent and is a first-line treatment for various cancers, including breast cancer, lung cancer, and prostate cancer [6]. DTX promotes microtubule assembly and stabilizes microtubules, thus arresting cells in the G2/M phase of mitosis, inhibiting cell proliferation, and inducing apoptosis, ultimately leading to cell death [7]. However, DTX faces several challenges in clinical application. Firstly, DTX has a short half-life, requiring frequent dosing to maintain effective plasma drug concentrations, which increases the treatment burden on patients [8]. Secondly, DTX lacks tumor specificity, often causing a range of severe side effects, including neuropathy, alopecia, and stomatitis [9]. Additionally, DTX’s high hydrophobicity and low solubility necessitate the use of potentially toxic solubilizers for intravenous administration, which may lead to hypersensitivity reactions [10–12]. Long-term use of chemotherapeutic drugs can also result in tumor cell resistance, where initially responsive tumor cells gradually lose sensitivity to the drug, ultimately leading to treatment failure [13,14]. Therefore, there is an urgent need to develop novel therapeutic strategies for lung cancer that can overcome the limitations of current chemotherapy, enhance efficacy, and reduce adverse effects, thereby improving the prognosis of lung cancer patients.

Photothermal therapy (PTT) has been widely regarded as a promising strategy for the treatment of solid tumors due to its high specificity, minimally invasive nature, spatiotemporal controllability, and broad adaptability [15–17]. The efficacy of PTT relies on the use of photothermal agents (PTAs), which effectively induce tumor cell apoptosis through localized heat ablation when exposed to near-infrared (NIR) laser irradiation [18–23]. However, one of the primary challenges faced by PTT is the development of tumor resistance to hyperthermia. Studies have shown that during PTT, tumor cells release heat shock protein 70 (HSP70), and the increased expression of this protein enhances the thermal resistance of tumor cells, reducing their sensitivity to PTT, thereby significantly diminishing the therapeutic efficacy of PTT [24–27]. This mechanism may cause tumor cells to develop resistance to subsequent PTT treatments following initial therapy, ultimately reducing the overall effectiveness of the treatment. Nonetheless, the study found that hyperthermia induced by laser irradiation can increase cellular sensitivity and vascular permeability, thereby leading to promoted tumor uptake of drugs for enhanced chemotherapeutic effects while avoiding damage to noncancerous areas [28,29]. In other words, the therapeutic strategy of combining PTT with chemotherapy can kill cells by using laser irradiation to raise the temperature in tumor regions while also enhancing the efficacy of chemotherapy through the synergistic effect of hyperthermia. Consequently, the synergistic effect of chemotherapy and PTT opens new possibilities for cancer treatment [30,31].

Recently, the integration of nanotechnology into combined therapy has witnessed remarkable advancements. A variety of nanoplatform-based drug delivery systems have been engineered for the co-delivery of chemotherapeutic agents, PTAs, inhibitors, and enzymes [32–35]. Nanoplatforms have certain advantages in tumor treatment owing to their tumor-targeting capability mediated by the permeability and retention effect. Additionally, by engineering nanoplatforms with responsiveness to stimuli such as pH, enzyme, light, and magnetism, precise control over drug release can be achieved. Compared with traditional drug formulations, nanodelivery systems can reduce toxic side effects while achieving improved bioavailability and enhanced therapeutic efficacy. However, the current studies of nanoplatforms are confronted with challenges such as complexed composite system, intricate fabrication methodologies, and suboptimal biocompatibility [36].

**Fig. 1.**
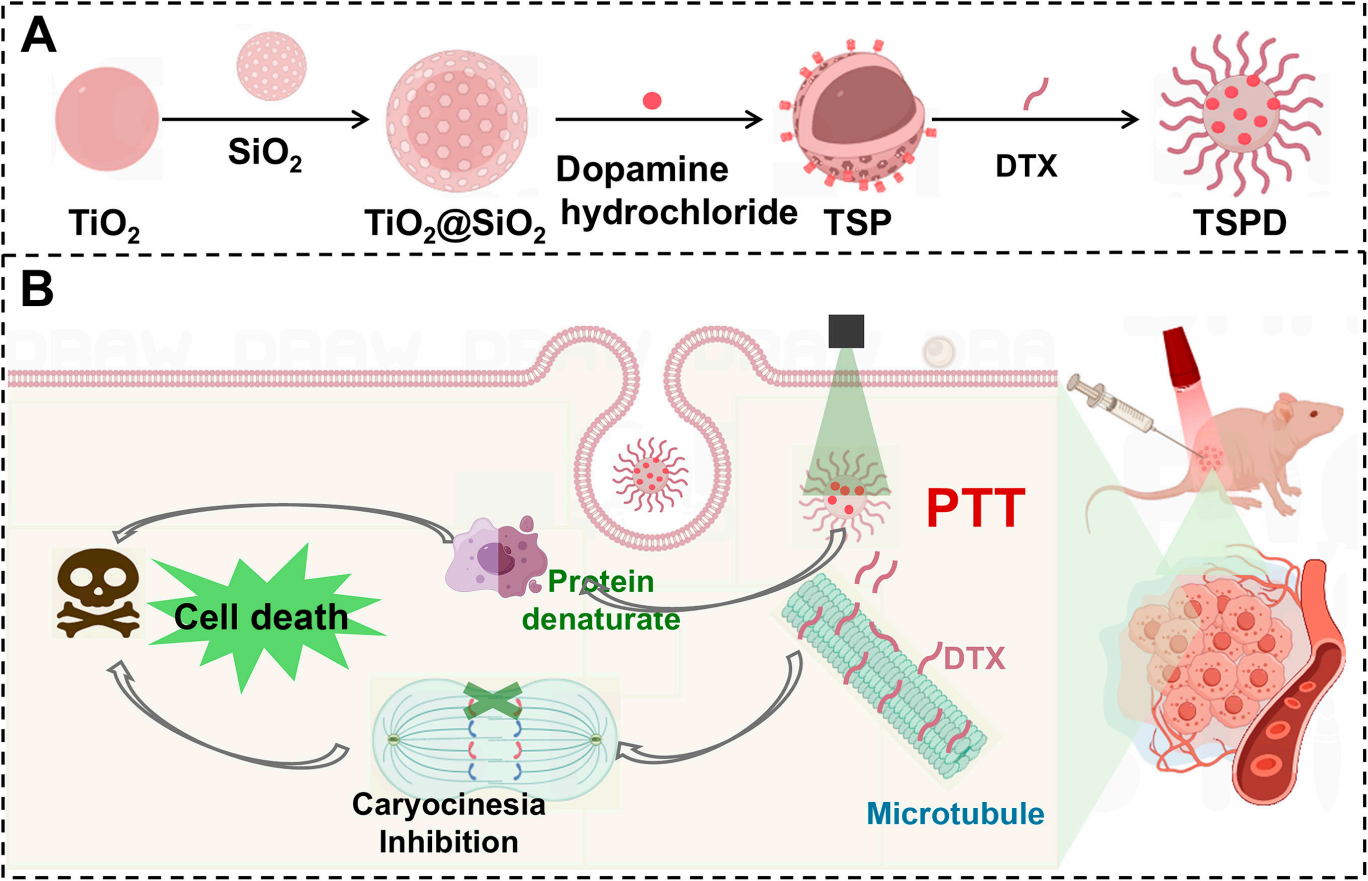
Schematic illustration of synergistic PTT/chemotherapy of lung cancer. (A) Synthetic route of TSPD. (B) Synergistic mechanism of combined PTT and chemotherapy for lung cancer treatment.

In this context, we have developed a novel drug delivery nanoplatform (TSPD) for the synergistic treatment of PTT and chemotherapy [37]. As illustrated in Fig. 1A, the platform utilizes titanium dioxide (TiO_2_) as a core template to produce porous silica-coated nanoparticles (TiO_2_@SiO_2_). Using an in situ polymerization approach, the PTA polydopamine (PDA) is coated onto the surface of TiO_2_@SiO_2_ to obtain TSP. DTX is then loaded onto TSP via hydrogen bonding and electrostatic interactions, resulting in the drug delivery nanoplatform (TSPD). Upon internalization by cancer cells, TSPD releases DTX in response to the acidic endosomes, where DTX interacts with microtubules to inhibit cell mitosis and suppress tumor growth. Concurrently, TSPD, with its high photothermal conversion efficiency, generates localized heat under NIR irradiation, further inducing cell death [38]. Furthermore, TSPD has a longer tumor retention in vivo, thus enhancing the therapeutic effect. Therefore, due to the synergistic effects of chemotherapy and PTT, TSPD nanoplatform demonstrates excellent tumor suppression capabilities and holds broad potential for application in lung cancer therapy.

## Materials and Methods

### Materials

DTX was purchased from Chia Tai Tianqing Pharma (Shanghai, China). TiO_2_, ammonium hydroxide, tetrathoxysilane (TEOS), polyethylene glycol (PEG), dopamine hydrochloride, indocyanine green, absolute ethyl alcohol, phosphate-buffered saline (PBS), and saline were purchased from Macklin Reagent Company (Shanghai, China). Fetal bovine serum (FBS) was purchased from PAN; 1640 medium was purchased from Gibco (Burlington, USA); 0.05% trypsin–EDTA, penicillin–streptomycin solution, calcein-AM dye, and propidium iodide (PI) dye were purchased from Biosharp (Guangzhou, China); cell counting kit-8 (CCK8) was purchased from Abbkine (Wuhan, China); DIO was purchased from MCE (New Jersey, USA); and mitochondrial membrane potential (MMP) assay kit (with JC-1) was purchased from Elabscience (Wuhan, China). Live/dead assay kit was from Thermo Fisher (Shanghai, China).

### Preparation for TSPD

First, 0.5 g of TiO_2_ was dispersed in 100 ml of deionized (DI) water, followed by ultrasound for 30 min by ultrasonic cleaners (Ulangee UC-9000, China); then, the dispersed suspension liquid was centrifuged for 30 min in 2,000 rpm by high-speed centrifuge (Zooelab SH2160R, China) to obtain the supernatant. Second, 0.133 g of TiO_2_ was dispersed in 20 ml of deionized (DI) water, followed by the addition of 100 ml of ethanol. The mixture was sonicated for 20 min to achieve uniform dispersion. Subsequently, 2.5 ml of ammonium hydroxide and 2 ml of TEOS were dropped, the reaction persisted for 20 h at room temperature, and the sediment was collected by centrifugation (10,000 rpm, 30 min) and washed 3 times by ethanol and DI water. Then, the sediment was added in PEG solution (1 mg/ml) and stirred overnight to further improve the dispersibility and to obtain TiO_2_@SiO_2_. Third, the modified TiO_2_@SiO_2_ and dopamine hydrochloride (molar ratio = 3:1) were dispersed in DI water, and the pH was adjusted to 12 to 13 by ammonium hydroxide and stirred for 4 h. Finally, TSP and DTX (mass ratio = 2:1) were mixed and dispersed in water and stirred overnight in the dark at room temperature to obtain the drug-loaded nanoparticles TSPD.

### Characterization

The morphology of TiO_2_, TiO_2_@SiO_2_, TSP, and TSPD was characterized by scanning electron microscopy (SEM; Zeiss Gemini 300) and transmission electron microscopy (TEM; Talos F200×, FEI), and the accelerating voltage was 120 kV. The hydrated particle size of nanoparticles was determined using a BeNano 90 Zeta nanoparticle size and zeta potential analyzer. The spectrum of TiO_2_, TiO_2_@SiO_2_, TSP, TSPD, and DTX at 200 to 800 nm was determined using a Shimadzu UV-2600 ultraviolet (UV) spectrophotometer to detect the uploading rate and the stability of TSPD in bovine serum protein.

### Photo-thermal conversion

Concentration-dependent photothermal effect: TSPD solutions at varying concentrations (1, 2, and 3 mg/ml) were irradiated with an 808-nm laser at a power density of 1 W/cm² for 5 min. Power density-dependent photothermal effect: TSPD solution (3 mg/ml) was subjected to 808-nm laser irradiation at different power densities (0.6, 0.8, 1.0, 1.2, and 1.4 W/cm²) for 5 min. Photothermal stability test: TSPD solution (2 mg/ml) was exposed to 808-nm laser irradiation (1 W/cm^2^) for 4 consecutive heating/cooling cycles. Each cycle consisted of laser irradiation for 5 min followed by natural cooling for 5 min without laser exposure. The temperature changes were recorded by an infrared imager (Thermo Scientific Nicolet iS20).

### In vitro experiments

Cytotoxicity was detected by CCK8 assay. Briefly, different cells were inoculated in 96-well plates at a density of 1 × 10^4^ cells per well and incubated separately at 37 °C, 5% CO_2_ for 12 h. After removing the previous layer of medium, fresh medium containing the samples was added and incubated for 24 h. To further investigate the cytotoxicity of TSP and TSPD, after 1 h of incubation, absorbance of cell viability was measured at 450 nm by a multi-plate reader (BioTek Synergy4, BioTek, USA).

For calreticulin AM/PI staining studies, cells were washed with PBS after treatment, stained with calreticulin AM/PI, and imaged with an inverted fluorescence microscope (IX71, Olympus, Japan). For apoptosis, 5 × 10^4^ cells per well were inoculated in 24-well plates, then 100 μg/ml TSPD was added, the 808-nm laser was given 1 W/cm^2^ for 5 min before treatment, and incubation was continued for 24 h. The medium was removed, and the cells were washed with PBS for 2 min, 100 μl of staining working solution was replaced in each well, and staining was performed under light protection for 20 min. Cells were washed with 1× PBS and then observed under a confocal laser scanning microscope (CLSM; TCS SP8, Germany), set to 10× objective (490 ± 10 nm). Live (yellow-green fluorescence) and Dead (red fluorescence) were observed. Moreover, the cell apoptosis induced by TSPD was measured by a flow cytometer (Beckman DxFLEX, USA).

To evaluate the intracellular uptake of DTX and TSPD, A549 and H460 cells were inoculated into 24-well plates, cultured overnight, and then incubated with the addition of fluorescein isothiocyanate (FITC)-conjugated DTX and TSPD administered in a time-graded gradient; incubated with 5 μg/ml of 4′,6-diamidino-2-phenylindole (DAPI) solution protected from light for 15 min; and washed 3 times with PBS. DIO solution (5 μg/ml) was added for 15 min, washed 3 times with PBS, and finally directly observed with a fluorescent confocal microscope.

To test the changes of cell MMP, A549 and H460 cells were cultured with TSPD at different incubated concentrations for 24 h. Then, the cells were stained with JC-1 and imaged on a CLSM.

### Animal model

Nude mouse was purchased from Guangdong Medical Laboratory Animal Center. All animal experiments were performed according to the guidelines approved by the Animal Ethics and Welfare Committee of the Southern Medical University. A549 cells (2 × 10^6^) suspended in 100 μl of PBS were subcutaneously injected into the 4- to 6-week-old mouse at the right axilla to establish the tumor model of animal.

### In vivo distribution of TSPD

Nude mouse (*n* = 3) was used for in vivo biodistribution studies of TSPD. In the experiments, each mouse was injected with PBS solution, pure Indocyanine Green (ICG), and TSPD in the tail vein. Real-time imaging was then performed in a dark room using an in vivo imaging system (IVIS AVPro-1023, BLT, China), and a series of fluorescent images were collected separately after injection. Mice were dissected at the 24-h time point, and fluorescence images of each organ (tumor, kidney, lung, spleen, liver, and heart) were collected. The fluorescence intensity of each group was recorded.

### In vivo photo thermography and tumor therapy

The mouse model of lung cancer was constructed, and when the tumor grew to about 100 mm^3^, mice were divided into 5 groups, 3 mice in each group. The control group was intratumorally injected with PBS with NIR treatment. Other groups were divided into TSPD (31.87 mg/kg), DTX (12.75 mg/kg), TSP + NIR (19.125 mg/kg), and TSPD + NIR (31.87 mg/kg). Mice in the group with NIR were treated with 808-nm laser irradiation (1.5 W/cm^2^) for 5 min after intratumor administration of the drug for 20 min. Body weights were recorded, and tumor volumes were determined every 2 d. After 14 d, the mice were over-anesthetized and executed, and tumors, serum, and organs were collected for further studies.

### Western blotting

Protein was extracted from tumors. After quantification, protein sample loading buffer was added and samples were boiled for 10 min at 95 °C. For sodium dodecyl sulfate–polyacrylamide gel electrophoresis (SDS-PAGE), 30 μg of total protein was loaded onto 10% PAGE (Servicebio). After protein was transferred onto polyvinylidene fluoride (PVDF) membranes (0.45 μm, Millipore-Sigma), membranes were blocked with 5% defatted milk for 30 min at room temperature and then incubated with primary antibody (anti-HSP70 antibody, 1:1,000) at 4 °C overnight. Blots were then incubated with horseradish peroxidase-conjugated anti-rabbit secondary antibody (Thermo Fisher Scientific), and data were recorded using chemiluminescent substrate (Servicebio, SCG-W2000).

### Histology and biochemistry analysis

On day 14 after sacrifice, heart, lung, liver, spleen, kidney, and tumors were taken and fixed with 4% paraformaldehyde, embedded in paraffin, and sliced. The tissue sections, at least 3 per mice, were subjected to hematoxylin and eosin (H&E) and immunohistochemical staining. All the images were obtained under a Nikon Eclipse E100 microscope.

### Immunofluorescence staining

For immunofluorescence staining, tumor issue sections were soaked in 3% bovine serum albumin at room temperature for 30 min after dewaxing, antigen regaining, and permeabilization. Sections were stained with mouse anti-CD31 primary antibody (dilution 1:200) and Alexa Fluor 488-conjugated goat anti-mouse secondary antibody (dilution 1:200) for marking CD31. Sections were stained at 4 °C overnight. The nucleus was then stained with DAPI. Images were observed and recorded by a confocal microscope (Zeiss, Germany).

## Results and Discussion

### Synthesis preparation and characterization of TSPD

**Fig. 2.**
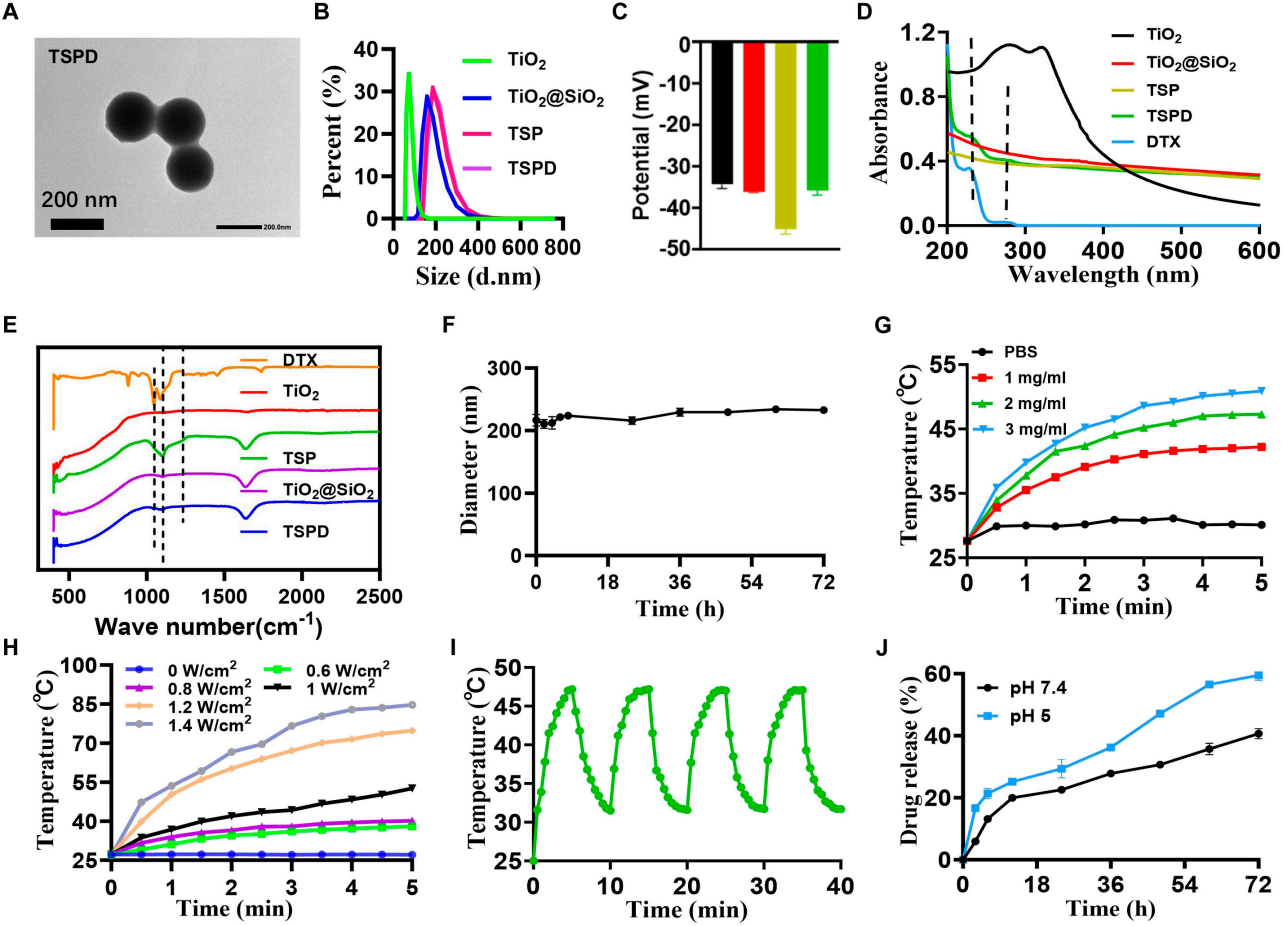
Characterization of nanoparticles. (A) TEM image of TSPD. (B) DLS profile of TiO_2_, TiO_2_@SiO_2_, TSP, and TSPD. (C) Zeta potential of TiO_2_, TiO_2_@SiO_2_, TSP, and TSPD (black: TiO_2_, red: TiO_2_@SiO_2_, yellow: TSP, green: TSPD). (D) UV–Vis spectra of TiO_2_, TiO_2_@SiO_2_, TSP, TSPD, and DTX. (E) FT-IR spectra of TiO_2_, TiO_2_@SiO_2_, TSP, TSPD, and DTX. (F) Size variation of TSPD in 10% FBS solution within 72 h. (G) Temperature variation curves of TSPD at different concentrations under 808-nm laser irradiation (1.0 W/cm^2^). (H) Temperature variation curves of TSPD (3 mg/ml) under 808-nm laser irradiation with different power densities. (I) Time-resolved photothermal oscillation profile of TSPD under periodic 808-nm laser irradiation. (J) Drug release profile of TSPD under different pH.

The synthesis process of the photothermal nanoplatform (TSPD) is illustrated schematically in Fig. 1A. TEM images provided in Fig. 2A and Fig. S1 reveal that TiO_2_@SiO_2_, TSP, and TSPD nanoparticles all exhibit well-defined spherical morphology, characterized by uniform particle size and good dispersion compared to the irregular and heterogeneous TiO_2_. The average particle size of TiO_2_@SiO_2_, TSP, and TSPD nanoparticles ranged from approximately 150 to 200 nm. Additionally, dynamic light scattering (DLS) measurements demonstrated that the hydrated size of the nanoparticles increased after PDA and drug loading. Specifically, the original size of 182 nm for TiO_2_@SiO_2_ increased to approximately 213 nm in the final TSPD nanoplatform (Fig. 2B). This change in size indicates successful incorporation of the drug within the nanoplatform matrix. As shown in Fig. 2C, the zeta potential analysis revealed that the zeta potentials of TiO_2_, TiO_2_@SiO_2_, TSP, and TSPD were all below −30 mV. Such low zeta potential values are advantageous, as they contribute to reduced cytotoxicity, enhancing the biocompatibility of the nanoplatform for potential therapeutic applications.

Further confirmation of the drug loading within TSPD was achieved through UV–visible (UV–Vis) spectroscopy and Fourier transform infrared (FT-IR) spectroscopy. Characteristic absorption peaks of the drug (DTX) at 228 and 273 nm were observed within the UV–Vis spectrum of TSPD, while the FT-IR spectrum showed that the peak of flexural oscillation of -CH at 1,040 cm^−1^ in DTX appeared at 1,045 cm^−1^ in TSPD, confirming the successful encapsulation of DTX into the nanoplatform system (Fig. 2D and E). The drug loading efficiency was quantified at 41.1%, based on a standard curve generated from known DTX concentrations (Fig. S2). Moreover, the stability of the TSPD nanoplatform in FBS was evaluated. The TSPD in 10% FBS solution showed a constant particle size distribution within 72-h incubation (Fig. 2F). In addition, as shown in Fig. S3, the absorbance peak at 230 nm exhibited only minimal fluctuations over time, with no remarkable wavelength shift. These findings indicated that the TSPD nanoplatform maintained their structural integrity and stability. This stability is crucial for ensuring reliable performance in subsequent in vitro and in vivo experimental settings. These results highlight the robust design of the TSPD system, positioning it as a promising candidate for further therapeutic exploration.

To assess the photothermal properties of TSPD, we monitored the temperature changes in a solution containing TSPD. Under 808-nm laser irradiation (1 W/cm^2^), the temperature of the material solution at a concentration of 1, 2, and 3 mg/ml rapidly increased, reaching 39.2, 42.4, and 45.1 °C within 2 min and further rising to 42.3, 47.3, and 51.5 °C within 5 min, respectively, while the temperature of PBS hardly changed (Fig. 2G). After laser irradiation with different power densities (0, 0.6, 0.8, 1, 1.2, and 1.4 W/cm^2^), the temperature of TSPD (3 mg/ml) showed a power dependence change and the amplitude of temperature rise was increased with the increase of laser power density (Fig. 2H). Additionally, the photothermal stability of TSPD was evaluated, demonstrating consistent performance over time (Fig. 2I). These results indicate that TSPD exhibits promising potential as a nanoscale PTA for tumor thermotherapy.

The drug release profile of TSPD was examined under different pH conditions, as illustrated in Fig. 2J. At pH 5, TSPD demonstrated a drug release rate of 59.5% over 72 h. In contrast, at neutral pH 7.4, the release rate was lower at 40.6%. This difference in release rates is likely due to the protonation of the amino group on the PDA under acidic conditions, which disrupts the interaction between the PDA and the DTX, resulting in accelerated drug release. The acidic environment not only promotes swelling of the nanoplatform but also enhances water penetration, leading to a higher drug release rate in acidic conditions compared to neutral pH. Furthermore, these findings suggest that DTX is more likely to be released in the endosomes, where the pH is as low as 5.0 to 6.0, rather than during systemic circulation, which could enhance drug availability at the tumor site and reduce potential side effects.

### Cell uptake and in vitro combination cell therapy of TSPD

**Fig. 3.**
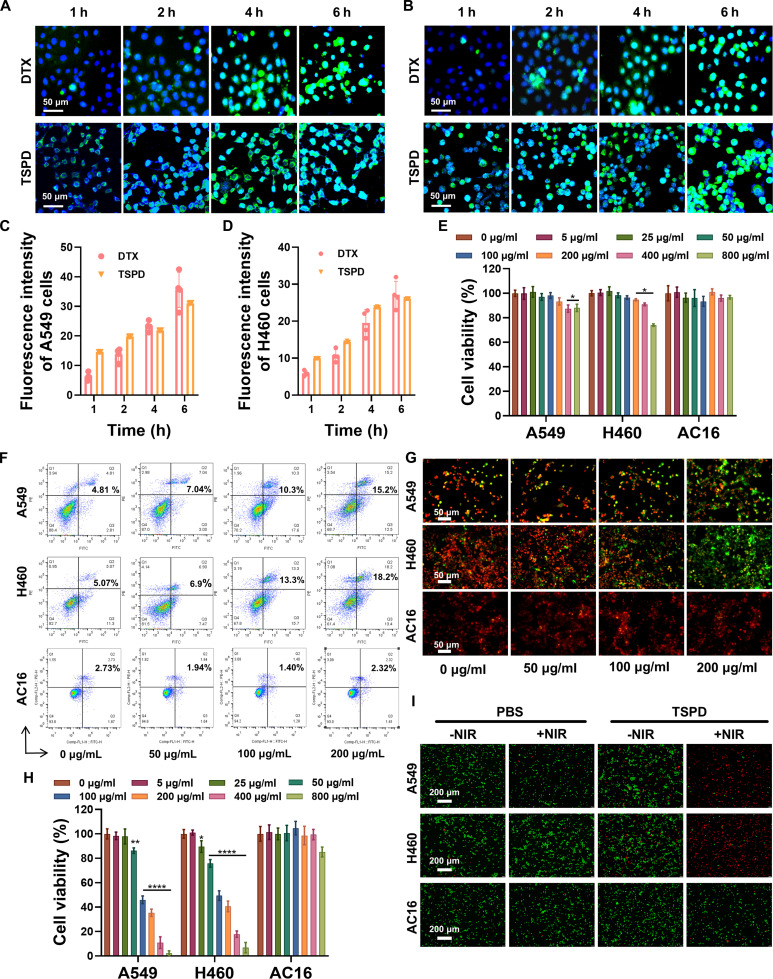
Effects of nanoparticles on cellular level. (A and B) CLSM images of DTX and TSPD uptaken by (A) A549 and (B) H460 cells. (C and D) Quantitative fluorescence analysis of fluorescence intensity emitted by FITC conjugated with DTX and TSPD from (C) A549 and (D) H460 (scale bar, 50 μm). (E) CCK8 assay of AC16, A549, and H460 cells treated with different concentrations of TSPD. (F) Apoptosis of AC16, A549, and H460 cells after treatment by TSPD in different concentrations. (G) Variations of MMP in AC16, A549, and H460 cells treated with TSPD under different concentration (scale bar, 50 μm). (H) CCK8 assay of different concentrations of TSPD with laser. (I) Live/Dead fluorescence images of AC16, A549, and H460 cells after TSPD treatment at indicated concentrations (scale bar, 200 μm). *P < 0.05, **P < 0.01, ***P < 0.001, ****P < 0.0001.

The in vitro cytotoxicity of TSP was assessed using the CCK8 assay in human lung cancer cells (A549 and H460) and myocardial cells (AC16) (Fig. S4). Remarkably, TSP exhibited negligible cytotoxicity in both cancer and normal cells, even at concentrations as high as 800 μg/ml, indicating excellent biocompatibility, making it a suitable candidate for biomedical applications. Intracellular uptakes of DTX and TSPD were investigated by co-incubating the cells with FITC-labeled DTX or TSPD. As shown in Fig. 3A to D, time-dependent green fluorescence from FITC was observed accumulating in A549 and H460 cells adjacent to DAPI-stained nuclei in both the DTX- and TSPD-treated groups within 6 h. In either cell, there is no significant difference between the 2 groups, indicating effective cellular uptake of TSPD and demonstrating that the nanoscale formulation did not markedly alter the cellular internalization behavior. In contrast, negligible fluorescence signal of FITC was observed in AC16 cells, which suggests that the cellular uptake of DTX and TSPD was limited (Fig. S5).

The therapeutic effect of TSPD on cells was initially assessed using the CCK8 assay. A statistically remarkable difference in A549 cell viability was observed compared with the blank control group when the concentration of TSPD was above 400 μg/ml (Fig. 3E). Additionally, TSPD at concentrations ≥200 μg/ml also inhibited the viability of H460 cells (Fig. 3E). Conversely, TSPD showed no significant effect on the viability of cardiomyocytes AC16, which might be attributed to their lower uptake of TSPD (Fig. 3E). Additionally, apoptosis was evaluated by flow cytometry, with Annexin V-FITC/PI staining revealing a concentration-dependent increase in cell apoptosis in the lung cancer cell lines A549 and H460, while the apoptosis rate of the cardiomyocyte cell line AC16 did not change obviously (Fig. 3F). MMP was subsequently evaluated. A concentration-dependent depolarization was observed in A549 and H460 cells, whereas AC16 cells exhibited no remarkable reduction in MMP under equivalent conditions (Fig. 3G). The reduction in MMP is an early event in the apoptotic cascade. Combined with the CCK8 and flow cytometry results, it is suggested that TSPD promotes apoptosis in lung cancer cells without causing severe cardiac toxicity. Furthermore, in vitro PTT was assessed through CCK8 (Fig. 3H) and live/dead staining assays (Fig. 3I). These assays demonstrated prominent cytotoxic effects against lung cancer cells under NIR irradiation compared to control groups, confirming the potent PTT effect of TSPD on cancer cells. Western blot experiments were also carried out to investigate the expression of HSP70 protein (Fig. S6). Exposure to TSPD plus 808-nm laser irradiation markedly elevated intracellular HSP70 levels in A549 and H460 cells, peaking at 0.5 W/cm^2^. By contrast, HSP70 expression in AC16 cells remained almost unchanged under all treatment conditions. These results suggested that TSPD exhibits a marked photothermal effect on lung cancer cells under 808-nm laser irradiation, with no notable PTT effect on cardiomyocytes.

### Biological distribution after injection of TSPD

**Fig. 4.**
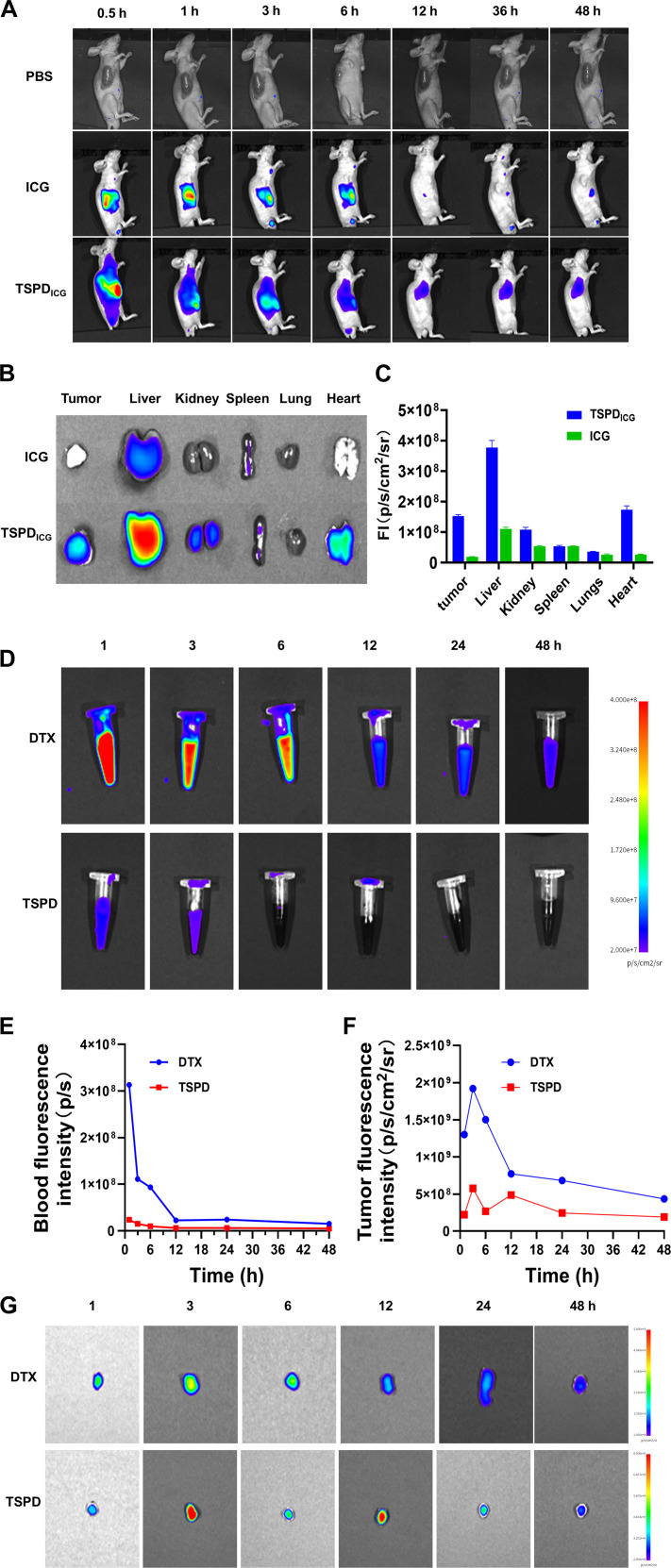
Distribution and metabolism of TSPD in A549-bearing mice. (A) Fluorescence imaging of A549-bearing mice at 0.5, 1, 3, 6, 12, 36, and 48 h after injection of PBS, ICG, and TSPDICG. (B) Ex vivo fluorescence imaging of tumor and important organs in TSPDICG and ICG groups at 48 h after injection. (C) Statistical fluorescence intensity of tumors and major organs in TSPDICG and pure ICG at 48 h after injection. (D) Serum fluorescence imaging and (E) corresponding fluorescence intensity of A549-bearing mice at 1, 3, 6, 12, 24, and 48 h after injection of Cy7-labeled DTX and TSPD groups. (F) Ex vivo tumor fluorescent intensity and (G) images of tumors extracted from A549-bearing mice at 1, 3, 6, 12, 24, and 48 h after injection of Cy7-labeled DTX and TSPD groups.

To investigate the biodistribution and tumor-targeting capabilities of TSPD, fluorescence imaging was conducted following the tail vein injection of ICG-labeled TSPD (TSPDICG) into mice. Whole-body fluorescence imaging over a 48-h period post-injection, comparing PBS, free ICG, and TSPDICG groups, revealed distinct distribution patterns (Fig. 4A). In the free ICG group, fluorescence intensity increased over time and was primarily localized in metabolic organs, such as the liver and kidneys. In contrast, the TSPDICG group exhibited highlighted fluorescence accumulation at the tumor site, with sustained detectability over the observation period. At 48 h post-injection, the mice were sacrificed, and major organs (tumor, heart, liver, spleen, lung, and kidneys) were harvested for ex vivo analysis. Fluorescence biodistribution studies demonstrated a preferential accumulation of TSPDICG at the tumor site, in stark contrast to the free ICG group, which showed more dispersed organ distribution (Fig. 4B). Further quantification of fluorescence intensity in the tumors and major organs revealed markedly higher TSPDICG retention within the tumor compared to the ICG group and most other organs at 48 h post-injection. This pronounced tumor targeting and retention of TSPDICG underscores its potential for enhanced PTT efficacy and precise tumor inhibition (Fig. 4C).

Meanwhile, the difference in the retention time of DTX and TSPD in tumors was compared. Cy7-labeled DTX and TSPD were administered via intravenous injection. Subsequently, blood and tumor tissue samples were collected at 1, 3, 6, 12, 24, and 48 h after injection for fluorescence intensity analysis. Serum fluorescence analysis showed that DTX remained in the bloodstream for 6 to 12 h, while TSPD maintained relatively low fluorescence levels in the blood (Fig. 4D and E). Ex vivo fluorescence imaging of tumors at different time points revealed that DTX was rapidly metabolized after entering the tumor tissue. In contrast, TSPD exhibited a slower metabolic trend and was retained in the tumor tissue for an extended period (Fig. 4F and G). These results indicated that, compared with the small-molecule drug DTX, TSPD has a longer retention time in tumor, which may contribute to enhanced therapeutic efficacy.

### In vivo antitumor properties and toxicity assessment of TSPDICG

**Fig. 5.**
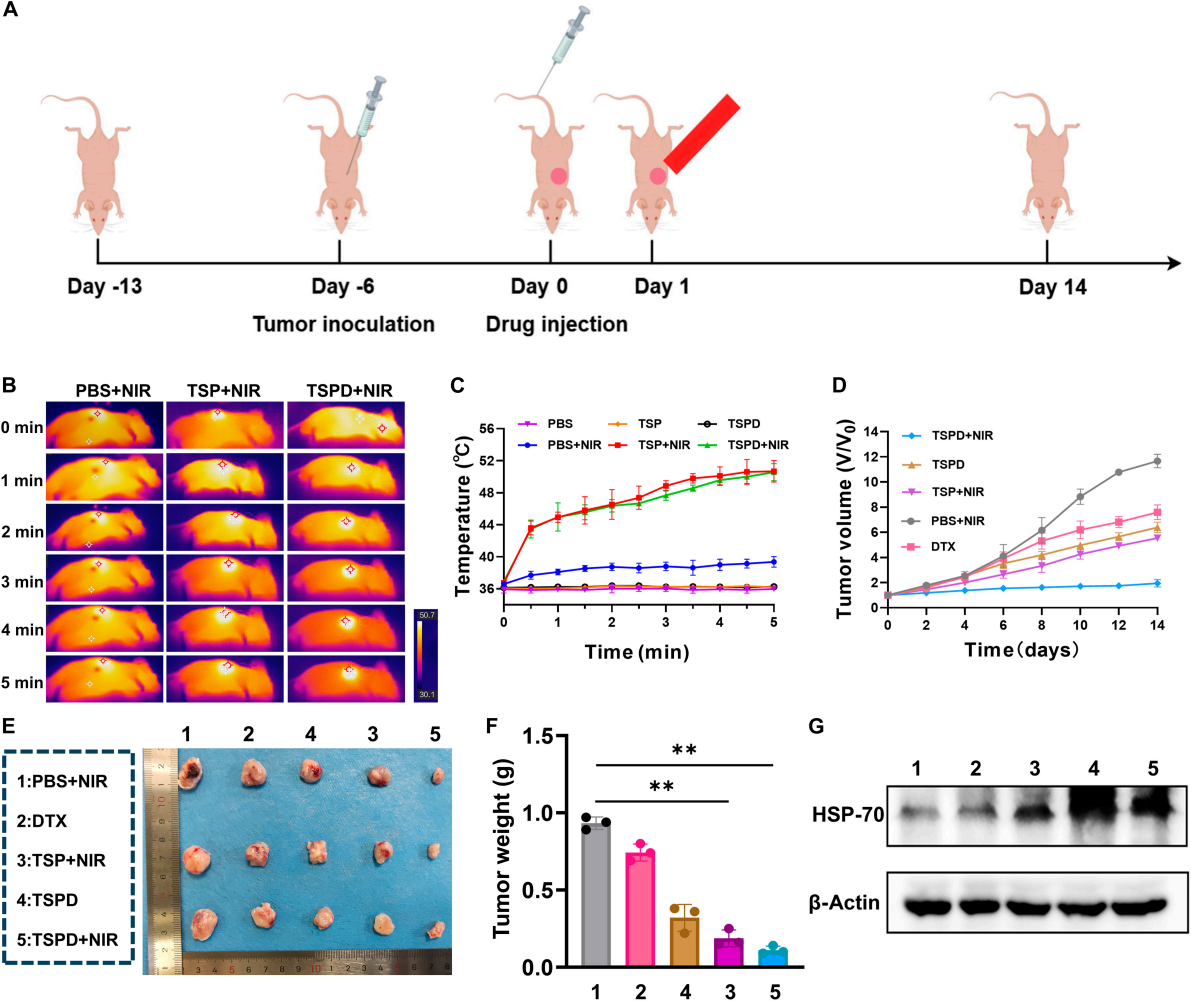
Effects of nanoparticles on animal level. (A) Schematic overview of the A549 mouse model experimental design. (B) Photothermal imaging of modeling mouse treated with PBS, TSP, and TSPD plus NIR. (C) Temperature variation curve of mice with different treatments. (D) Plots of the variation in tumor volumes during the therapy. (E) Representative images of extracted tumors from mice under different treatment. (F) Weight profile of tumors demonstrated in (E). (G) Expression of HSP70 in extracted tumor tissue from mice under different treatment.

To demonstrate the in vivo antitumor efficacy of TSPD, its therapeutic effects were evaluated in A549 lung tumor mouse model, with the experimental design outlined in Fig. 5A. Building on the outstanding in vitro results, we assessed PTT potential of TSPD following the successful establishment of the lung tumor model mice. Twenty minutes after nanoplatform injection, real-time temperature changes in the mice were recorded using a thermal imaging camera (Fig. 5B and Fig. S7). The tumor temperature rapidly increased to 43.5 °C within 30 s and gradually rose to approximately 50 °C after 5 min of 808-nm laser irradiation (Fig. 5C). Throughout the treatment period, the body weight of the mice remained stable across all groups, except for the DTX-treated group, which showed a temporary decline, likely due to the side effects of DTX (Fig. S8D). This further demonstrated the excellent biosafety of the nanoplatform formulations, with or without NIR laser exposure (Fig. S8D). Notably, as shown in Fig. 5D, tumor growth in the PBS + NIR and DTX groups was rapid, while the tumor growth in the TSPD and TSP + NIR groups was remarkedly slowed. In the TSPD + NIR group, tumor growth was almost completely halted. After 14 d of treatment, the mice were sacrificed, and the tumors were excised. The tumor size in the TSPD + NIR group was visibly smaller compared to other groups (Fig. 5E), which was consistent with the tumor weight measurements (Fig. 5F). Additionally, the expression levels of HSP70 were examined by Western blot (Fig. 5G). The TSPD + NIR group showed the highest expression level of HSP70, indicating that TSPD can indeed generate heat under light irradiation and induce a PTT effect.

**Fig. 6.**
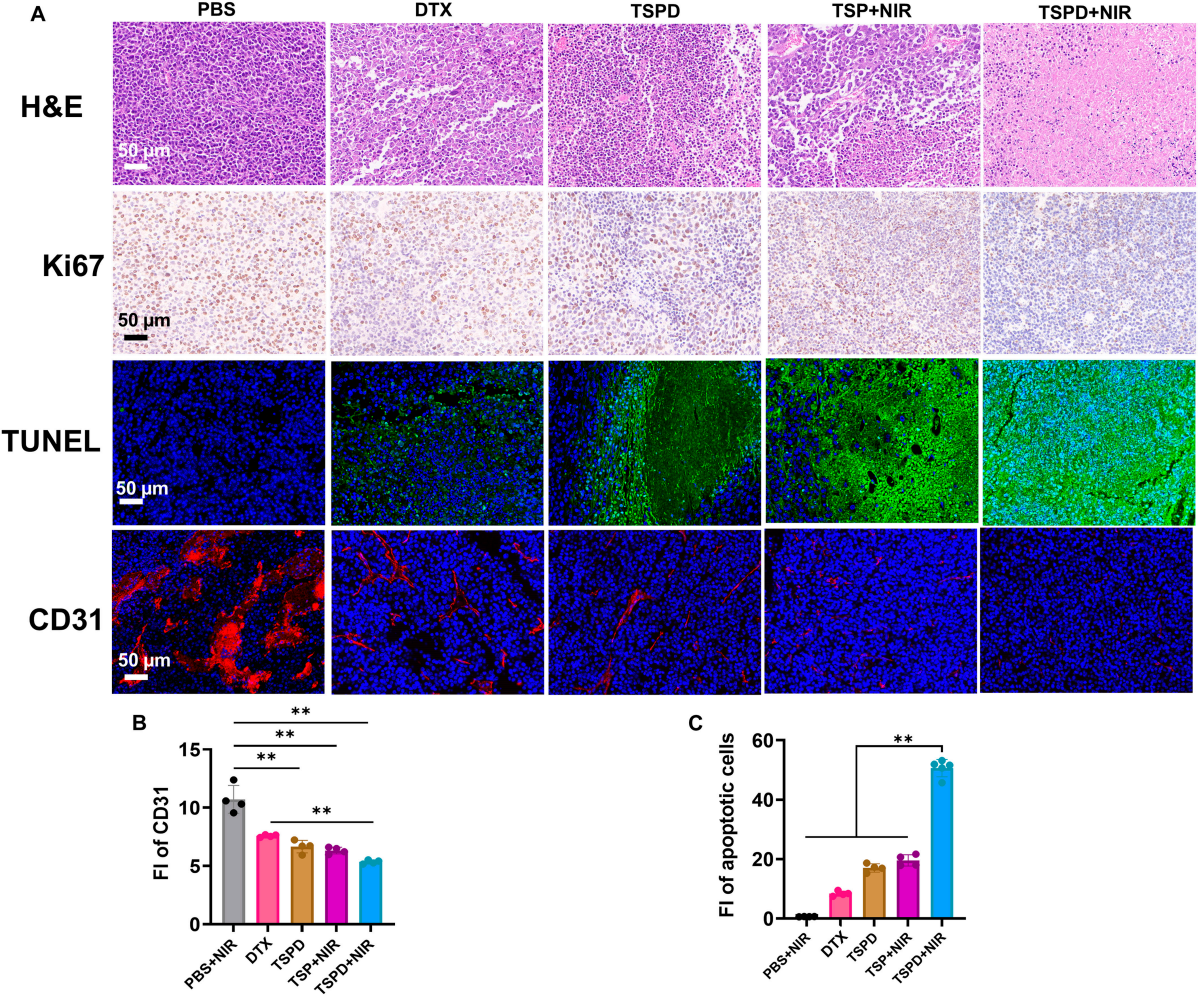
Staining tumor tissues extracted from mouse model treated under different conditions. (A) H&E, TUNEL, and IHC staining of Ki67 and CD67 in different groups. Scale bar, 50 μm. (B and C) Quantification of CD31 (B) and apoptotic cells in Ki67 (C) in the 5 groups. Scale bar, 50 μm. *P < 0.05, **P < 0.01, ***P < 0.001.

Subsequent histological analyses, including H&E, Ki67, and immunohistochemical (IHC) staining, were conducted to further elucidate the therapeutic outcomes. In line with the tumor growth data, tumor sections from the TSPD + NIR group displayed the most extensive areas of necrosis. Ki67 staining, a widely used marker for assessing cell proliferation, revealed a marked reduction in proliferating cells in the TSPD + NIR treatment group (Fig. 6A). Furthermore, CD31 staining, an indicator of tumor angiogenesis, showed a remarkable reduction in endothelial cell markers in the TSPD + NIR group, confirming the treatment’s effectiveness in inhibiting tumor angiogenesis (Fig. 6A and B). TUNEL (terminal deoxynucleotidyl transferase-mediated deoxyuridine triphosphate nick end labeling) staining further demonstrated elevated levels of apoptosis in the TSPD + NIR group compared to the other groups, underscoring the enhanced cytotoxic effects of this combination therapy (Fig. 6A and C). Importantly, no obvious lesions were observed in major organs across all treatment groups (Fig. S9), indicating the safety of the drug-loaded nanoplatform and the absence of significant side effects during treatment.

To comprehensively evaluate the toxicity of TSPD, varying concentrations (0, 1, 5, 10, and 20 mg/ml) were intravenously administered to healthy mice. After 21 d, the mice were sacrificed, and their major organs were examined for histological changes. H&E staining revealed normal cellular morphology and no significant lesions in any of the organs tested, confirming the excellent biocompatibility and safety profile of TSPD (Figs. S8B and C and S10).

## Conclusion

In summary, we successfully developed TSPD nanoplatform using a straightforward method, demonstrating excellent injectability, biocompatibility, photothermal properties, and notably prolonged retention at the tumor site. This nanoplatform prominently enhanced therapeutic efficacy against lung cancer through synergistic chemo/photothermal therapy. Notably, tumor volume in the TSPD + NIR group was significantly reduced, underscoring the effectiveness of the targeted combination therapy in improving resistance to DTX. These findings provide preclinical evidence that targeted chemo/photothermal therapy may outperform conventional treatments for lung cancer. The spatial and temporal control of drug release through TSPD targeting ensures precise tumor-specific delivery, reducing the adverse effects associated with DTX. This study positions nanoplatform as a noninvasive, precise therapeutic strategy, providing a valuable tool for targeted chemo/photothermal therapy in translational lung cancer research.

## Data Availability

Data will be made available on request.
