# Supplementary material for: Near-Infrared Responsive Nanoplatform for Synergistic Photothermal Therapy/Chemotherapy of Lung Cancer
Source: Biomater Res. 2026 Mar 6;30:0319. doi: 10.34133/bmr.0319 (PMC12963644; doi:10.34133/bmr.0319)
Supplement: Supplementary 1 — Figs. S1 to S10 [file bmr.0319.f1.docx]

**Supplementary Materials:**

**Near-Infrared (NIR) Responsive nanoplatform for Synergistic Photothermal/ Chemo-Therapy of lung cancer**

Kangqi Ren_2†_, Jian Wang^2†^, Lei Xue^1^, Jieyu Liu^2^, Jun Li^1^, Degang Liu^1^, Qiang Yang^1^, Jiangzhou Peng_1*_

† These authors contributed equally to this work.

^1*^Department of Thoracic Surgery, The Third Affiliated Hospital of Southern Medical University, Guangzhou, 510630, China.

^2^Department of Thoracic Surgery, The Shenzhen People’s Hospital, The Second Clinical Medical College of Jinan University, Shenzhen, 518020, China.

**^*^Corresponding author(s)**: ^1*^Department of Thoracic Surgery, The Third Affiliated Hospital of Southern Medical University, Guangzhou, 510630, China.

1. **mail(s)**: [Jiangzhou83@163.com](mailto:Jiangzhou83@163.com).


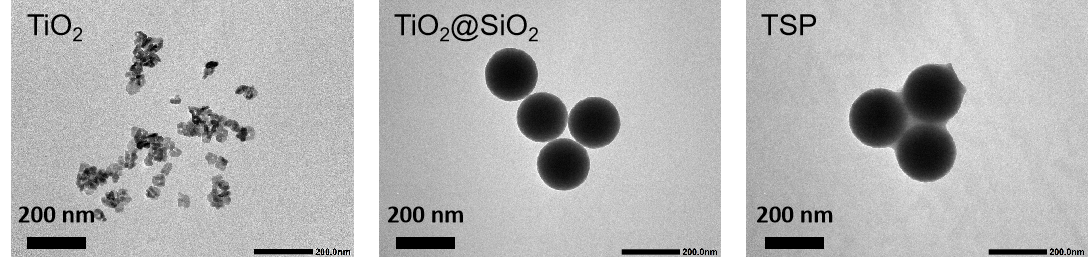


Fig. S1 TEM images of TiO_2_, TiO_2_@SiO_2_ and TSP.


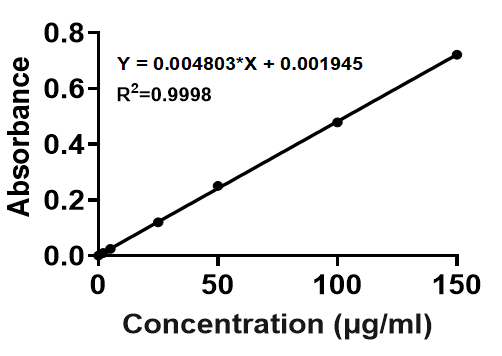


Fig. S2 The standard curve of DTX.


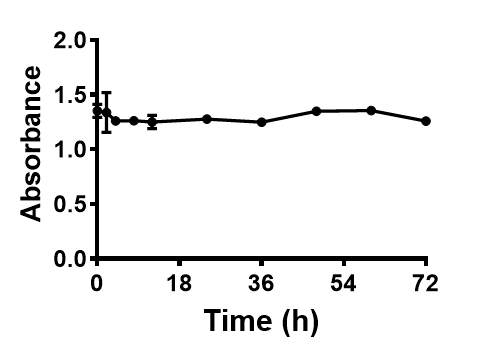


Fig. S3 The UV-Vis absorbance at 230 nm of TSPD in 10% FBS solution.


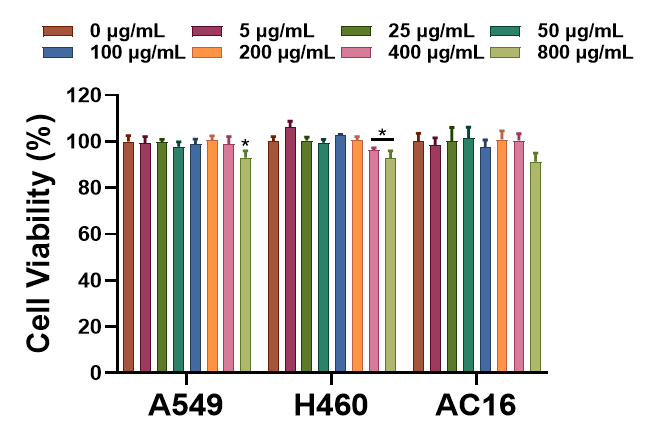


Fig. S4 A549, H460 and AC16 cell viability after incubation with TSP.


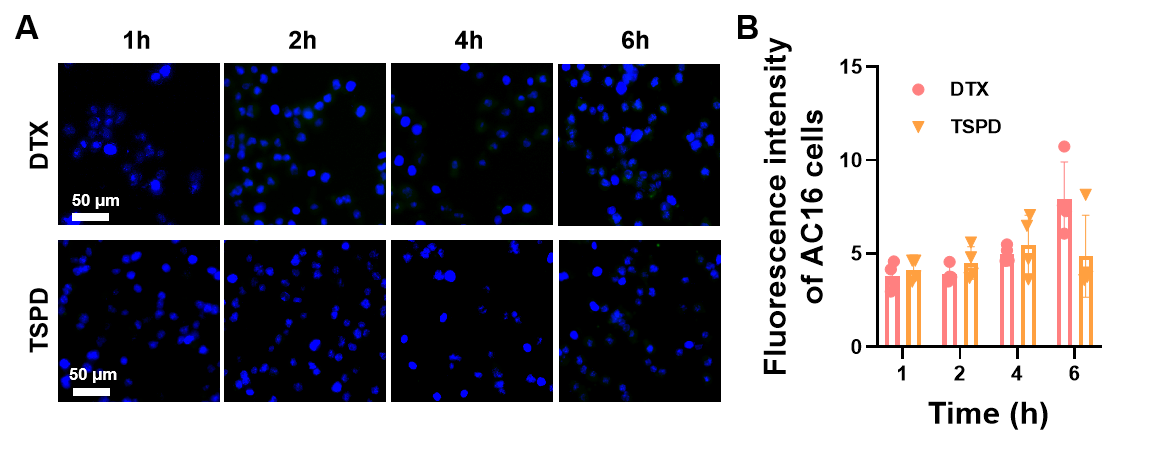


Fig. S5 (A) AC16 cell uptake of DTX and TSPD were detected using CLSM and (B) corresponding quantitative fluorescence analysis.


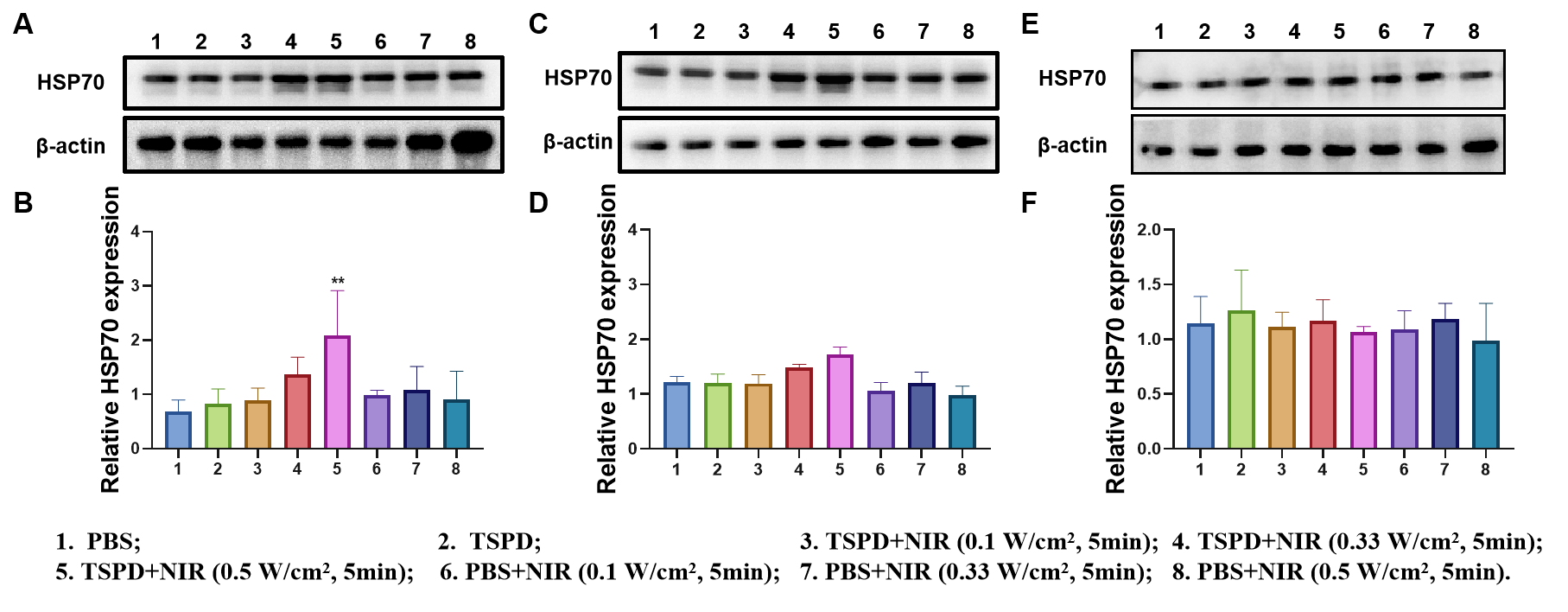


Fig. S6 (A) Expression of HSP70 detected by Western blot in A549 cell. (B) The quantitative analysis of Western blot results in (A). (C) Expression of HSP70 detected by Western blot in H460 cell. (D) The quantitative analysis of Western blot results in (C). (E) Expression of HSP70 detected by Western blot in A16 cell. (F) The quantitative analysis of Western blot results in (E).


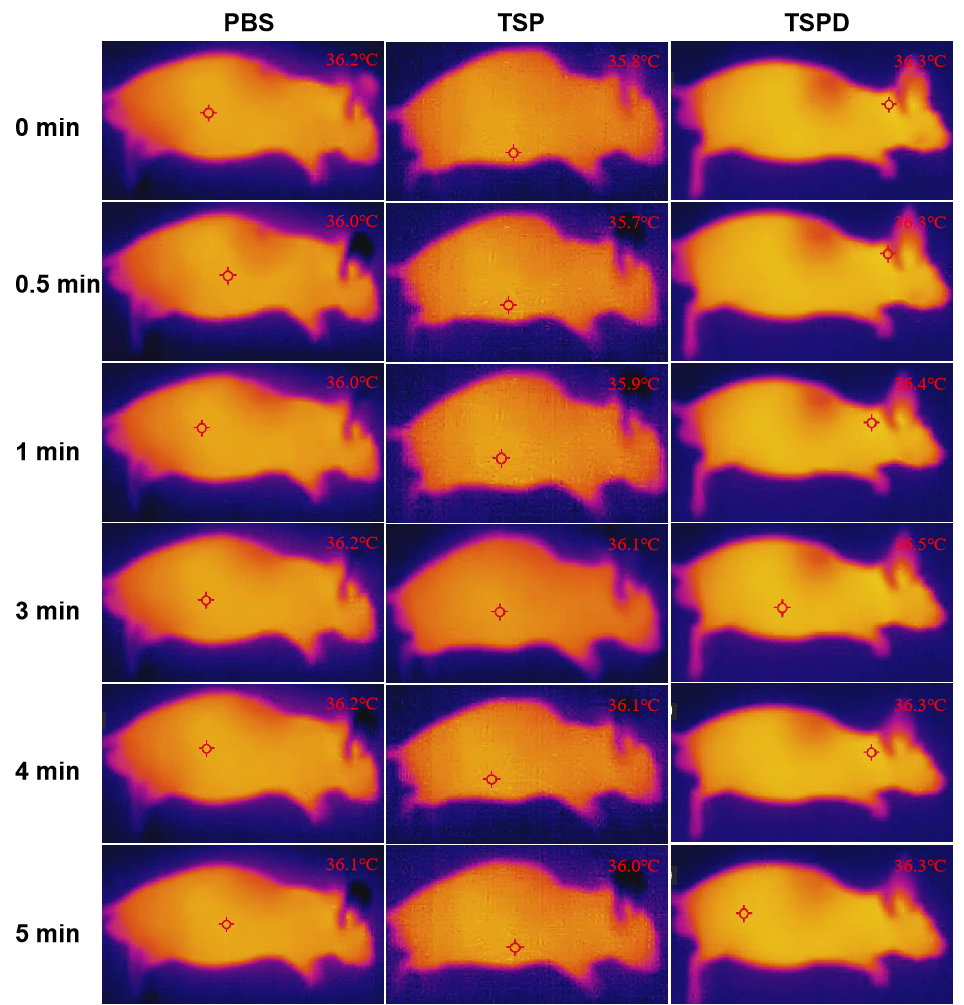


Fig. S7 The photothermal imaging of modeling mouse treated with PBS, TSP and TSPD.


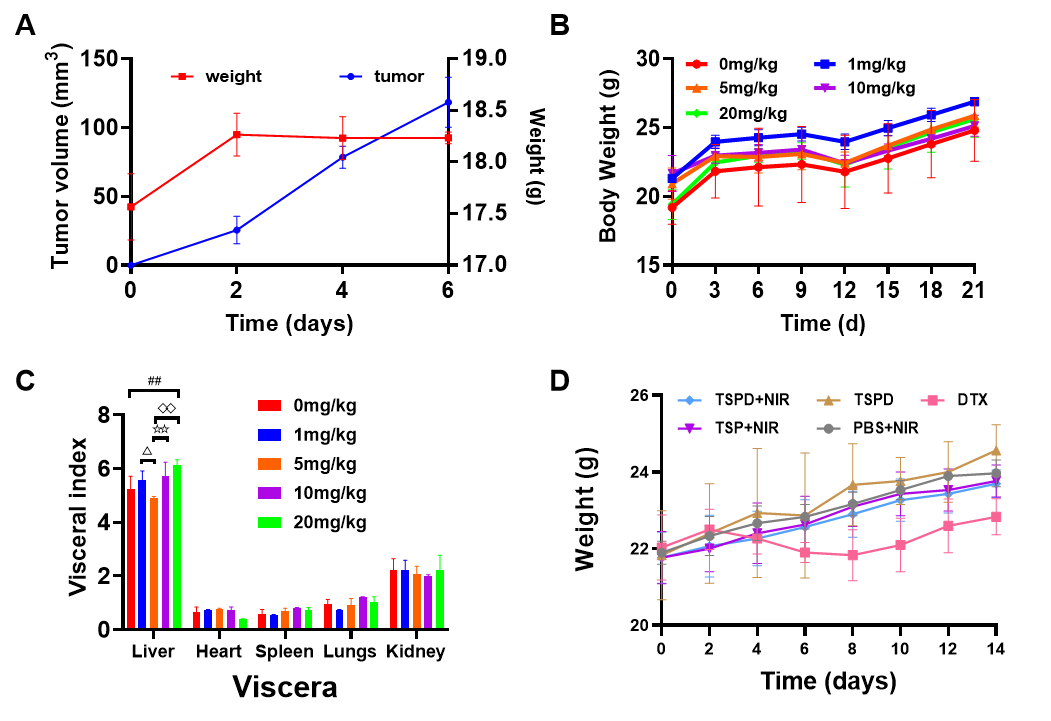


Fig. S8 (A) The growth of tumor volume and body weight after inoculation within 6 d. (B) The weight index of treatment by different concentration of TSPD. (C) The organ index of treatment by different concentration of TSPD. (D) The weight variations of mice after the treatment.


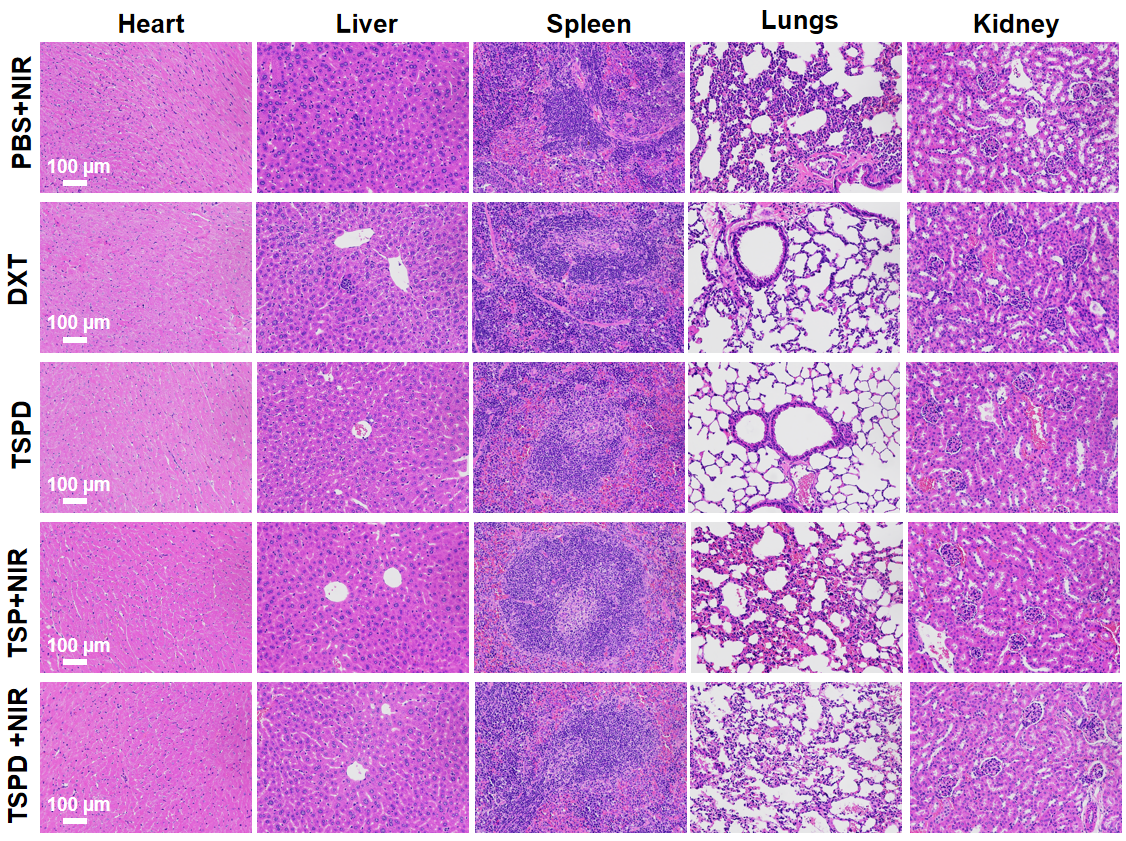


Fig. S9 H&E staining images of major organs from mouse models after different treatments.


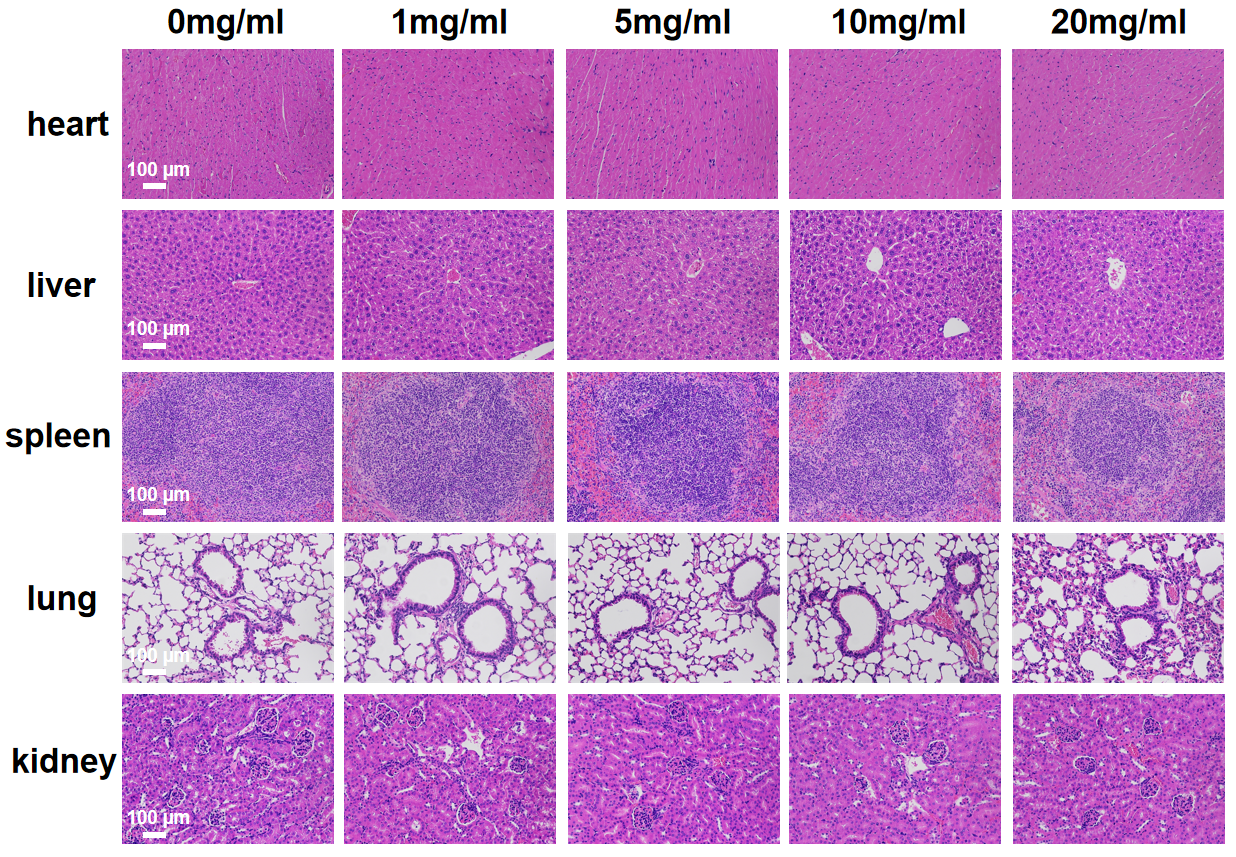


Fig. S10 H&E staining images of major organs from mice administrated with TSPD at different concentrations.
